# Facilitating pragmatic skills through role-play in learners with language learning disability

**DOI:** 10.4102/sajcd.v64i1.187

**Published:** 2017-07-26

**Authors:** Fareeaa Abdoola, Penelope S. Flack, Saira B. Karrim

**Affiliations:** 1 Discipline of Speech-Language Pathology, University of KwaZulu-Natal, South Africa

## Abstract

**Background:**

Role-based learning involves the process whereby learners acquire skills, knowledge and understanding through the assumption of roles within real-life settings. Role-play holds potential as an effective learning strategy for children; however, there is limited research on the use of role-play as a therapy method within the field of speech-language pathology. Children with language learning disability (LLD) typically present with difficulties in social communication, which can negatively affect their social and academic achievement.

**Aim:**

The aim of this study was to determine the effectiveness of role-play as a therapy approach targeting the pragmatic skills of stylistic variation and requesting for clarification in learners with LLD.

**Method:**

The use of combined positivist and interpretivist paradigms allowed for the implementation of an embedded mixed methods design. An experimental pretest-posttest design was implemented. Eight participants, who were learners with a diagnosis of LLD, were purposefully selected. Data collection was conducted over five phases, utilising the Clinical Evaluation of Language Fundamentals (4th Ed.) Pragmatics Profile, discourse completion tasks, session plans and session records. Quantitative data were analysed using descriptive statistics and were supplemented by qualitative data from session records.

**Results:**

Results revealed improvements in stylistic variation and requesting for clarification post role-play intervention, with minimal changes in the control group. Limitations of the study have been reported for consideration when interpreting results.

**Conclusion:**

Role-play as a therapy approach targeting two pragmatic skills, stylistic variation and requesting for clarification, was found to be beneficial for learners with LLD. Recommendations for the implementation of role-play as a therapy approach were made.

## Introduction

One often sees children playing ‘make believe’ and taking on the role of a schoolteacher, mother or doctor. Children generally have experience of taking on the role of another person in a different situation from a young age. Literature suggests that role-play is a natural method adopted by children to learn, as all children engage in some form of socio-dramatic play (Goldstein & Cisar, 1992; McSharry & Jones, 2000). In the field of speech-language pathology, therapists seek out evidence-based methods of learning to facilitate language development in children who require such intervention. Role-play holds potential as an effective method of learning for children (Clarke & Wales, 2005; Greenwood, Horton & Utley, 2002; Killen, 2006; Mason, 2006; Purvis, 2008). Its effectiveness as an approach to targeting pragmatic skills in children with language learning disability (LLD) was investigated in this research study.

### Literature review

#### Role-play

Role-play can be defined as a technique using simulated communication scenarios to elicit specific or spontaneous responses (Purvis, 2008). Clinically, simulation aims to provide experience in a safe and secure environment through the imitation of reality (Theodoros, Davidson, Hill & MacBean, 2010). In role-based learning settings, the learner is a participant in the setting that simulates a real-life scenario. The role of the therapist in this setting is that of a facilitator who guides and creates learning opportunities (Killen, 2006; Oliver, Harper, Hedberg, Wills & Agostinho, 2002). The implementation therefore requires purposeful preparation on the part of the facilitator to develop scenarios that provide learning opportunities in accordance with the objectives (Oliver et al., 2002).

In this study, literature on the use of role-play as a learning strategy has been sourced primarily from the field of education and applied to speech-language pathology. In the field of speech-language pathology, role-play has been used along with other methods to target social communication skills; however, there is no study that investigates the effectiveness of role-play itself as an intervention approach (Gerber, Brice, Capone, Fujiki & Timler, 2012). A recent study found that theatre-based intervention with children with autism spectrum disorder resulted in improvements in social cognition, social interaction and social communication (Corbett et al., 2015). This randomised trial made use of peer-mediated learning and acting in a theatre context to target social competence and has provided initial evidence supporting theatre-based intervention (Corbett et al., 2015). This relates closely to the use of role-play as a therapy approach as it required the participants to take on the role of another in a given scenario, thereby providing a naturalistic context for learning social skills.

#### Pragmatics

The term ‘pragmatics’ is typically used to refer to the ways in which speakers and listeners use language in social interaction (Goldstein, Kaczmarek & English, 2002). ASHA (2015) defines pragmatics as the system combining language components (phonology, morphology, syntax and semantics) to generate functional and socially appropriate communication. This definition illustrates the complex nature of pragmatics, as it relies on and comprises of multiple language skills (Adams, 2002). Another aspect of pragmatics, which adds to its complex nature, is that it is culturally and linguistically diverse (ASHA, 2015). In the South African context, caseloads are largely multilingual and multicultural; speech-language therapists must therefore be aware of the cultural differences in pragmatics when assessing and providing intervention to individuals with social communication deficits (McLeod, 2014; Perry, 2012).

Two specific pragmatic skills were selected and targeted in the role-play intervention for this study. These were requesting for clarification and stylistic variation (register). Requesting for clarification refers to making a request to repair or clarify the message when communication breakdown occurs. This involves identifying that you have not understood the message and then making the speaker aware that you have not received the message. A request for clarification can involve verbally telling the speaker that you do not understand, asking them to repeat themselves or even giving a non-verbal cue such as an enquiring look. As children develop language, they typically first learn to respond to requests for clarification from others around two years of age (Fletcher, O’ Toole & Fourie, 2015). However, as their language develops they learn to independently make requests for clarification at around four to five years of age (Fletcher et al., 2015).

Stylistic variation refers to the ability to shift from one register to another, according to the communication partner and context. For example, one would use an informal register while interacting with friends at break time, but will have to switch to a formal register if asked to meet with the principal or boss. Register is also context sensitive, as one may use a less formal register if speaking to the principal or boss at a social event, and a more formal register if speaking to the principal or boss regarding school or work. Children begin to appropriately alter their register from as early as 4 years of age (Paul, 2007).

#### Language learning disability

The Diagnostic and Statistical Manual of Mental Disorders, fourth edition, (DSM-IV) of the American Psychiatric Association (2000) provides acknowledged guidelines to establishing a diagnosis of a language disability. The DSM-IV defines learning disability as follows: ‘learning disorders are diagnosed when the individual’s achievement on individually administered tests of reading, mathematics or written expression is substantially below that expected for age, schooling and level of intelligence’. The DSM-V (American Psychiatric Association, 2013) introduces a change in terminology by referring to ‘specific language disorder’. This term combines the DSM-IV diagnosis of reading disorder, mathematics disorder, disorder of written expression and learning disorder not otherwise specified. Children with LLD, therefore, fall under this category. In this study, the DSM-IV would have been utilised to diagnose participants.

Literature suggests that difficulties experienced by children with learning disabilities affect not only their academic performance, but also their ability to use language appropriately in social contexts (Funderburk, Schwartz & Nye, 2009; Hallahan & Kauffman, 2003; Vaughn, Elbaum & Boardman, 2001). It is imperative that these difficulties are addressed in intervention, as they have the potential to affect the individual’s ability to become an integrated member of society. Poor pragmatic skills can result in peer rejection, decreased likability and difficulty forming friendships (Cordier, Munro, Gillan & Docking, 2013). This in turn increases the risk of low self-esteem, long-term emotional difficulties and social isolation (Brinton & Fujiki, 2006). Effective approaches to address pragmatic difficulties are therefore necessary.

## Rationale

The rationale for this study stems from personal clinical experience and observation, where it was noted that children in a special needs classroom were more involved in the therapy session and more easily retained new vocabulary when role-play was used. This exemplified literature regarding learners requiring more explicit intervention that supports generalisation and provides immediate feedback (Greenwood et al., 2002). Investigation into role-play as a learning strategy dates back at least 30 years (Ladousse, 1987; Van Ments, 1983), where the use of role-play was found to be effective in the education context. A recent change in approach to teaching and learning strategies has seen a rise in focus given to constructivism and active learning. These concepts are based on the tenet that effective learning occurs when the learner is actively involved in the construction of knowledge, as opposed to receiving knowledge from a third party (Brady, 2004). Various studies in the field of education advocate for the use of role-play as an active learning strategy (Brady & Skully, 2005; Clarke & Wales, 2005; Killen, 2006; Yehuda, 2006). A problem often encountered by speech-language therapists is that of a lack of generalisation of therapy aims to contexts outside the therapy environment. Role-play allows the therapy context to closely approximate natural interactions, and therefore results in more functional outcomes and increased generalisation (Killen, 2006). Even though role-play is used in certain areas of speech-language pathology, there is sparse literature documenting its method of implementation and effectiveness.

## Methodology

### Aim and objectives

The aim of this study was to determine the effectiveness of role-play as a therapy approach targeting pragmatic skills in learners with LLD. The objectives focused on determining the effectiveness of role-play in improving the two pragmatic skills being targeted to achieve the aim, that is, stylistic variation and requesting for clarification.

### Research design

The combined use of positivist and interpretivist paradigms allowed the researcher to logically analyse the research data, while still considering the holistic view through observation and interpretation (Coolican, 2004; Weaver & Olsen, 2006). This was achieved through the use of an embedded mixed methods design. Qualitative data were used to support quantitative data, in order to view a complete picture and achieve data triangulation. The dominant quantitative component made use of an experimental pretest-posttest design. This method controlled for many threats to internal validity, while showing that change occurred following the treatment.

**FIGURE 1:**
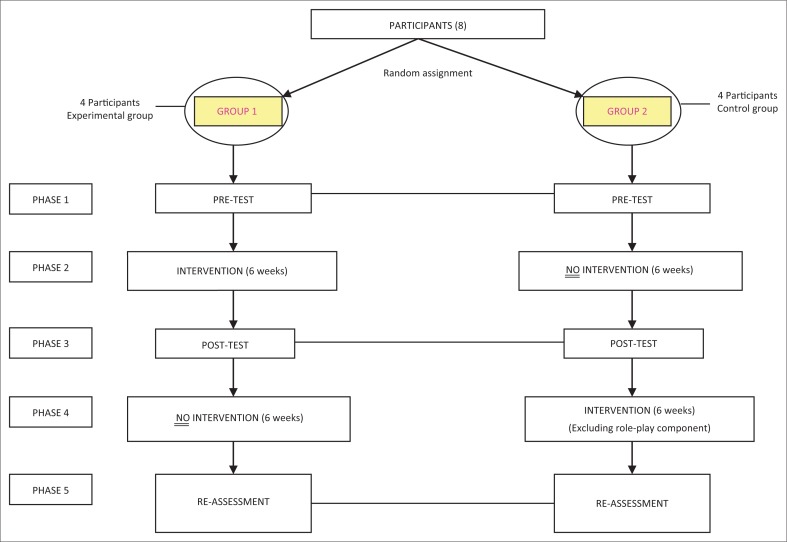
Structure of the experimental pretest-posttest design.

Eight participants, between the ages of 10 and 12, who were learners with a diagnosis of LLD, were purposefully selected (Appendix 1). All the participants attended a school for learners with special educational needs in eThekwini (KwaZulu-Natal) and resided in surrounding areas. All participants spoke English as their dominant language. Intervention focused on two specific pragmatic skills: Stylistic variation and requesting for clarification. Data collection was conducted over five phases (see Figure 1 below). Phase 1 involved pragmatic assessment of each of the participants. Phase 2 consisted of the role-play intervention being implemented with the experimental group. Phase 3 served as the posttest, and thus involved a reassessment of all of the participants (experimental and control group). An additional step was then added to the traditional pretest-posttest design. During Phase 4, the control group received intervention targeting the same pragmatic skills; however, no role-play was used. Thereafter, all participants received a final reassessment in Phase 5. This final step allowed the researcher to compare the effects of the intervention with and without the inclusion of role-play.

### Procedure

An assessment of each participant was conducted using the Clinical Evaluation of Language Fundamentals, fourth edition, Pragmatics Profile (CELF-4 PP) and discourse completion tasks (DCTs). Assessment was conducted through observation in various contexts (classroom and playground) and one-on-one interaction with the participants. When using pragmatic skills as an outcome measure, it is recommended that assessments allow for documentation across various contexts and communication partners (Gerber et al., 2012; Norbury, 2014). The DCTs were designed by the researcher based on research studies that made use of DCTs (Archer, Aijmer & Wichmann, 2012; Chen & Rau, 2013; Jernigan, 2007; Kasper & Dahl, 1991; Kasper & Roever, 2005; Martínez-Flor & Uso-Juan, 2011). The situations presented in the tasks all included the following information: The setting, social distance and social status (Alemi, Rasekh & Razanjad, 2014; Aufa, 2014; Chen & Rua, 2013; Kasper & Roever, 2005; Martínez-Flor & Uso-Juan, 2011). The scoring of the DCTs was conducted by a third party in an effort to avoid researcher bias.

The intervention phases of the study involved the implementation of group therapy. Literature provides limited guidelines for best practice in social skills group interventions (Reichow & Volkmar, 2010). Group therapy sessions were therefore designed and implemented by the researcher using guidelines for implementing role-play as a learning strategy described in literature (Brady & Skully, 2005; Cherif, Verma & Somervill, 1998; Clarke & Wales, 2005; Killen, 2006; Ladousse, 2004; McDaniel, 2000; Milroy, 1982, Yehuda, 2006). Each session comprised five components: introduction, narrative, discussion, role-play and reflection. The narratives were written by the researcher based on the criteria of a social story (Gray, 2000) (Appendix 2). Narratives were designed to be linguistically and culturally appropriate while addressing the target pragmatic skill. Interaction with the participants during the selection and assessment process allowed the researcher time to gauge information on the participant’s linguistic abilities and cultural background prior to formulating the narratives. The narrative served the purpose of providing the learners with a foundation on which to practice the skill (Duncan & Klinger, 2010; Stanton-Chapman & Snell, 2011).

A structured record form was completed for each participant following every group session (Appendix 3). The form allowed for documentation of the session in general, as well as record-keeping of each participant’s performance. The purpose of documentation was to achieve data triangulation and so that the variables (e.g. lack of interest of the participant) could be accounted for during interpretation and discussion of results.

### Data analysis

In keeping with the research design, the qualitative data were embedded in the quantitative data in the analysis and discussion of results. Integration of the two sets of data was conducted at the reporting level, using a weaving approach (Fetters, Curry & Creswell, 2013). This was achieved by first tabulating and presenting each set of data and thereafter integrating the results in a written analysis or narrative (Fetters et al., 2013). The qualitative data served to enrich, support (data triangulation) and provide explanations for the quantitative data. Because of the small sample size, quantitative data were analysed with the use of only descriptive statistics. Assessment scores were analysed by calculating and comparing the mean, standard deviation and gain in scores. The reliability of these descriptive statistic scores when using a small sample depends directly on the reliability of the pre and posttest scores (Salkind, 2010; Zimmerman, 2009). The purpose of these measures was simply to provide a means of drawing comparisons across phases and groups.

Analysis of results was separated into analysis of the experimental group and analysis of the control group. The initial step in the group analysis was to provide an overview of each participant’s response to the intervention; this allowed for the documentation of clinically significant findings that may be masked with statistical analysis of combined group scores alone (Adams, 2003). The second step was to conduct a statistical analysis of the groups’ pre- and post-assessment scores, and analyse this information in conjunction with qualitative data. This provided the information needed to conduct the last step of analysis. The last step involved comparison of the experimental and control group, in order to determine the effectiveness of role-play as a therapy approach targeting pragmatic skills (stylistic variation and requesting for clarification) in learners with LLD. For the purpose of this article, only the combined group scores and a comparison thereof are presented.

### Trustworthiness

Reliability in this study was ensured by the researcher administering the data collection instruments herself or himself, the utilisation of user-friendly data collection instruments, a portion of the assessment being conducted by a third party and by conducting a pilot study. Validity in this study was ensured by the use of an instrument that is based on literature and criterion referenced (CELF-4 PP) (Semel, Wiig & Secord, 2003), conducting a pilot study prior to the main study, and the use of a control group to provide as a comparative measure. Validity of results was also achieved by controlling for extraneous variables, for example, participants received no other intervention for pragmatic skills during the duration of the study.

## Results

The experimental group received intervention during Phase 2, with the pre- and post-intervention assessments occurring in Phases 1 and 3 (see Figure 1). Results from Phases 1 and 3 were analysed and presented in Tables 1 and 2 below.

**TABLE 1:** Experimental group: Statistical analysis of assessment scores (Phases 1 and 3).

Assessment measure	Phase1		Phase 3		Gain score	
		
	Mean	Standard deviation	Mean	Standard deviation	*n*	%
CELF-4 PP	118.50	7.681	129.50	10.472	11	9
DCT	13	7.165	16.50	4.359	3.5	26

CELF-4 PP, Clinical Evaluation of Language Fundamentals, fourth edition, Pragmatics Profile; DCT, discourse completion task.

**TABLE 2:** Control group: Statistical analysis of assessment scores (Phases 1 and 3).

Assessment measure	Phase1		Phase 3		Gain score	
		
	Mean	Standard deviation	Mean	Standard deviation	*n*	%
CELF-4 PP	128	6.272	128.50	4.796	0.5	0.39
DCT	10.75	3.862	10.75	5.560	0	0

CELF-4 PP, Clinical Evaluation of Language Fundamentals, fourth edition, Pragmatics Profile; DCT, discourse completion task.

With regard to the control group, results from Phases 1 and 3 were analysed first (Table 2), and thereafter results from Phases 3 and 5 were analysed (Table 3). The control group received no intervention during Phase 2 and received intervention excluding role-play (the session plan included only the introduction, narrative, discussion and reflection) in Phase 4.

**TABLE 3:** Control group: Statistical analysis of assessment scores (Phases 3 and 5).

Assessment measure	Phase3		Phase 5		Gain score	
		
	Mean	Standard deviation	Mean	Standard deviation	*n*	%
CELF-4 PP	128.50	4.796	128.75	7.411	0.25	0.19
DCT	10.75	5.560	17.75	0.500	7	65

CELF-4 PP, Clinical Evaluation of Language Fundamentals, fourth edition, Pragmatics Profile; DCT, discourse completion task.

Session records for each participant were reviewed and summarised in the table below (Table 4). Data from session records were used to supplement and support quantitative data findings.

**TABLE 4:** Summary of qualitative data as per session records.

Group	Participant	Motivation, participation and attitude	Progress noted
Experimental	1	Motivated to attend. Attentive and well behaved. Did not actively participate.	Improved understanding of target pragmatic skills (particularly requesting for clarification).
	2	Motivated to attend. Good participation. Provided peer support. Poor attention in three sessions.	Improved understanding of target pragmatic skills Progress noted from fourth session.
	3	Motivated to attend. Good participation. Active involvement. Provided peer support.	Improved understanding of target pragmatic skills. Progress noted from second session.
	4	Poor attention. Poor participation. Reported to be tired during six of the sessions.	Minimal improvement in understanding of target skill noted in Session 4. Increased understanding of both target skills noted from Session 8.
	5	Motivated to attend. Level of participation depended on how relatable the narrative was to her.	Improved understanding of target pragmatic skills. Progress noted from fifth session.
Control	6	Motivated to attend. Poor concentration. Disruptive to session.	Minimal improvement in understanding of target skills.
	7	Reluctant to attend. Good participation. Difficulty maintaining attention for duration of session.	Improved understanding of target pragmatic skills. Progress noted from fifth session.
	8	Motivated to attend. Poor attention and concentration. Minimal active participation.	Improved understanding of target pragmatic skills. Progress noted from eighth session.

Results from Phases 1 and 3 revealed that the average increase in the mean of the experimental group for both assessment measures was greater than that of the control group. The CELF-4 PP and DCT scores of the experimental group increased by an average of 11 and 3.5, respectively (Table 1), while the control group CELF-4 PP and DCT scores increased by an average of 0.5 and 0, respectively (Table 2). Improvements in the experimental group post-intervention were further supported by participant-specific data from session records (Table 4).

It was found that improvements were noted post-intervention for both requesting for clarification and stylistic variation. Requesting for clarification was targeted for the first six group sessions and stylistic variation was targeted during the second six group sessions. Improvements in both these skills were more apparent in performance on the DCT, which assessed them directly. It was found that participants were able to grasp and apply the concept of requesting for clarification more easily than stylistic variation. Even though participants were already familiar with the facilitator and the components of the session, they took longer to independently identify pragmatically appropriate and inappropriate behaviour with regard to stylistic variation. This may be because stylistic variation is context specific, and is therefore more cognitively and linguistically demanding. It was also found that generalisation occurred to untrained skills as well, which was more apparent in participants who were actively involved in group sessions (Participants 2 and 3).

Phases 3–5 of the study involved the control group receiving intervention, while the experimental group received no intervention. However, the control group received the intervention without the role-play. The purpose of this was to allow the researcher to compare the effects of the intervention with and without the role-play component, thus establishing if it is in fact the use of role-play that is effective. Results indicated that the experimental group presented with a higher average increase in scores on the CELF-4 PP post-intervention (Mean increase: 11), as compared with the control group (Mean increase: 0.25). However, the control group presented with a higher average increase in score on the DCT assessment compared with the experimental group. The low increase in DCT scores of the experimental group appears to be as a result of two of the participants already achieving a high score on the DCT assessment pre-intervention. Analysis of session records revealed that the control group did make progress in therapy without the role-play component (Table 4). This progress appears to have reflected in their post-intervention DCT assessment. The progress, however, did not reflect in the post-intervention pragmatic profile assessment. The reason for this could be that progress was made in the therapy context, but did not generalise, and was therefore not observed when completing the profile during classroom and break time observation.

## Ethical consideration

Ethical clearance was obtained from the University of KwaZulu-Natal, Humanities and Social Sciences Research Ethics Committee (Reference number: HSS/0334/014M). Informed consent was obtained, as learners became participants in the study only if they provided verbal consent and their parent or caregiver provided written consent. Right to privacy or confidentiality was ensured by assigning a pseudonym to each participant for reference throughout the study and by not revealing any information about the participants and organisations involved. The raw data collected from the study are stored in password-protected electronic files and will be kept for at least 5 years. Only the researcher and supervisors have access to these data.

## Discussion

This study aimed to determine the effectiveness of role-play as a therapy approach to target stylistic variation and requesting for clarification in learners with LLD. It was found that there is limited research into effective methods of addressing pragmatic difficulties of learners with LLD. The need for such research is evident in the fact that learners with LLD typically present with difficulties in social communication (Funderburk et al., 2009; Hallahan & Kauffman, 2003), which negatively affects their social relationships, inclusion and quality of life (Diken, 2014). Results from both quantitative and qualitative data revealed that improvements in stylistic variation and requesting for clarification were observed post role-play intervention in the experimental group, with minimal changes in the control group.

Role-play as a therapy approach targeting pragmatic skills (stylistic variation and requesting for clarification) in learners with LLD was found to have a number of benefits that supported its effectiveness. These included that participants displayed increased interest and involvement when role-play was used; participants reported to enjoy the ‘acting’ and would enthusiastically decide who should play which role when it came to the role-play component of the session. During intervention with the control group (which excluded role-play), even learners who participated well began to lose interest and concentration before the end of the session. Research shows that if learners are not actively involved in the process of knowledge acquisition, they are less likely to make the necessary connections that make learning meaningful (Cuthrell & Yates, 2007). Role-play also allowed for peer learning to take place. It was found that stronger participants supported weaker participants, by offering prompts, modelling and giving examples and suggestions. This was noted during the role-play and reflection components of the group session. Participants responded well to support from their fellow learners and generally responded to the prompt or suggestion. Lastly, it was found that skills learnt appeared to generalise to outside the therapy context and were maintained after a period of 6 weeks of no intervention. Qualitative analysis of the CELF-4 PP (Semel et al., 2003) pre and post-intervention revealed that generalisation occurred to untrained skills as well as target skills. Role-play creates a ‘real-life’ type context for the learner (Killen, 2006; Van Ments, 1999), and practising a skill in realistic contexts increases the likelihood of generalisation of the target skill (Stewart, Carr & LeBlanc, 2007). Parent and teacher input during the pre- and post-assessment process, in the form of questionnaires or rating scales, is recommended to validate findings in future studies (see Gerber et al., 2012).

Limitations of the approach were also identified. During role-play intervention, it was found that the responsibility of creating an environment that supports active learning relies on the facilitator. The facilitator needs to maintain a role that is supporting and flexible (Killen, 2006; McDaniel, 2000), while ensuring support is graded according to the needs of each participant. The effectiveness of the intervention is therefore dependent on the facilitators’ skill in supporting child-centred learning. Role-play intervention can also be time consuming to plan and implement, and it relies on learner participation.

**FIGURE 2:**
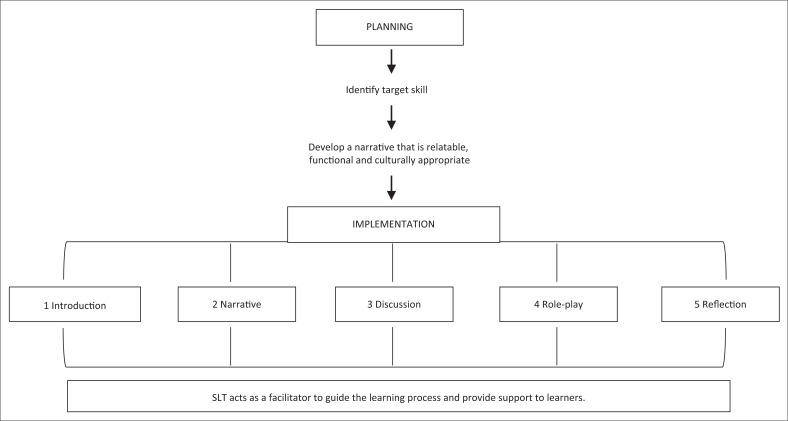
Recommended steps for planning and implementing role-play as a therapy approach.

It can be concluded that all of the components in the session plan in role-play-based therapy were found to be necessary and beneficial for effective implementation of the intervention. However, further research is needed to fine-tune each component, in order to achieve the best outcomes. The recommendations for the implementation of role-play as a therapy approach derived from this study has been consolidated and presented in Figure 2 below. The figure presents the steps recommended in planning and implementation of a session, as well as the facilitator’s role in the process.

### Clinical implications

Speech-language therapists should seek evidence-based methods for addressing the pragmatic difficulties of learners with LLD. The findings of the study can be used to inform clinical practice and decision-making when implementing role-play intervention. Therapists should be aware of and implement all the components of a role-play session. Appropriate knowledge of the process of active learning will ensure maximum benefit being derived from role-play. Therapists should also be aware of using narratives that are relatable, functional and culturally appropriate. Strategies to support and ensure the participation of weaker learners, such as peer learning, should be supported during role-play intervention.

### Limitations

The following were identified as limitations of the study: The small sample size (eight participants) in this study limits the extent to which results can be generalised; all the participants were from the same school; participants were limited to learners whose dominant language is English; assessments of pragmatic skills pre and post-intervention were conducted only in relation to the school context; a portion of the assessment was conducted by the researcher (researcher bias); and random assignment of participants resulted in unequal distribution of abilities in the control and experimental groups. Furthermore, data collection was conducted over a period of two and a half months; participant maturation could therefore be a possible confounding variable.

### Research implications

Future research in the area of role-play as a therapy approach should investigate its effectiveness in targeting pragmatic skills in learners with LLD, using a larger sample size, and with learners with other developmental communication disorders. Given the multilingual, multicultural clientele found in South Africa (Jordaan, 2011), and the cultural nature of pragmatic skills, the effectiveness of role-play as a therapy approach targeting pragmatic skills in learners with LLD, who are English second language learners, should be researched. The scope of the use of role-play can also be expanded by exploring its effectiveness across age groups, pragmatic skills and other areas of language. The development of standardised guidelines and principles for planning and implementation of role-play intervention will ensure evidence-based practice.

## Conclusion

Role-play is an active learning strategy that closely mimics natural interactions, and therefore results in improved generalisation of skills (Killen, 2006). The combined use of positivist and interpretivist paradigms allowed the researcher to logically analyse the research data, while still considering the holistic view through observation and interpretation (Coolican, 2004; Weaver & Olsen, 2006). This was achieved through the use of an embedded mixed methods design. Qualitative data were used to support quantitative data, in order to view a complete picture and achieve data triangulation. The method of implementing role-play intervention was sourced from education literature and was found to be effective in its use as an intervention approach in speech-language pathology. It can be concluded that in this study role-play was found to be an effective approach to target stylistic variation and requesting for clarification in learners with LLD. Role-play as an approach to intervention may therefore be the way forward in ensuring generalisation of pragmatic skills. However, results of the study should be interpreted with the limitations in mind. The results of this study have also identified further areas of research regarding the use of role-play as a therapy approach and provided therapists with recommendations to inform their clinical practice. The results of this study have laid the foundation for future research and implementation of role-play as a therapy approach in speech-language pathology.

## Appendix 1

**TABLE 1-A1:** Description of participants.

Participant code	CA (years)	Gender	Communication profile (as per school speech therapy file)	Intelligence quotient (IQ)
1	11; 0	Female	Age inappropriate receptive and expressive language. Particular deficits in auditory memory, following instructions, semantics and pragmatics.	IQ range 50–59
2	11;4	Male	Mild difficulties in receptive language. Poor expressive language, phonological awareness, and articulation difficulties.	IQ range 90–100
3	11;2	Female	Mild difficulties receptive language. Poor expressive language, phonological awareness and articulation difficulties.	IQ range 70–79
4	11;6	Male	Age inappropriate receptive and expressive language. Poor pragmatic skills (topic maintenance, eye-contact) Note: ADHD diagnosis.	IQ range 90–100
5	10;5	Female	Mild difficulties in receptive language. Age inappropriate expressive language. Poor phonological awareness skills.	IQ range 50–59
6	10;2	Male	Age inappropriate receptive and expressive language. Difficulty following instructions, poor auditory memory, poor phonological awareness, and poor pragmatic skills.	IQ range 50–59
7	11;6	Male	Mild difficulties in receptive language. Age inappropriate expressive language and pragmatics. Poor phonological awareness abilities.	IQ range 90–100
8	11;2	Male	Age inappropriate receptive and expressive language. Difficulty following instructions. Poor pragmatics (topic maintenance, requesting, understanding and use of non-verbal communication).	IQ range 50–59

ADHD, attention deficit hyperactivity disorder; CA, chronological age.

## Appendix 2

**TABLE 2-A2:** Example of narrative reflecting application of the principles of social stories.

Narrative	Criteria
Title: Kim learns to ask.	Teach with the title
Kim is a 10 year old girl who goes to Kings Primary School.	*Introduction*: Descriptive sentence (Who)
Kim’s class was helping the teacher clean the classroom.	*Body*: Descriptive sentence (Where)
The teacher told Kim to dust the table cloth.	*Body*: Descriptive sentence (What)
Kim could not hear the teacher properly because the class was making noise.	*Body*: Descriptive sentence (What)
The teacher was angry at Kim because she did not listen and dust the tablecloth.	*Body*: Perspective sentence
Kim learnt that if she does not hear what someone said, she should ask.	*Conclusion*: Directive sentence

## Appendix 3

Session record form

Date:

Time:

Venue:

Group therapy session no.:

Pragmatic skill targeted:

Description of therapy environment:

Researcher’s personal reflection:

Researcher’s assessment of session

Recommendations for next session

PARTICIPANT

General conduct:

Motivation and participation in session:

Performance in session:

Progress noted (if applicable):
